# Shorter Telomeres May Mark Early Risk of Dementia: Preliminary Analysis of 62 Participants from the Nurses' Health Study

**DOI:** 10.1371/journal.pone.0001590

**Published:** 2008-02-13

**Authors:** Francine Grodstein, Marieke van Oijen, Michael C. Irizarry, H. Diana Rosas, Bradley T. Hyman, John H. Growdon, Immaculata De Vivo

**Affiliations:** 1 Channing Laboratory, Department of Medicine, Brigham and Women's Hospital, and Harvard Medical School, Boston, Massachusetts, United States of America; 2 Department of Epidemiology, Harvard School of Public Health, Boston, Massachusetts, United States of America; 3 Department of Epidemiology, Erasmus Medical Center, Rotterdam, The Netherlands; 4 Department of Neurology, Erasmus Medical Center, Rotterdam, The Netherlands; 5 Glaxo-Smith Kline, Research Triangle Park, North Carolina, United States of America; 6 Massachusetts Alzheimer Disease Research Center, Massachusetts General Hospital, Department of Neurology, Harvard Medical School, Boston, Massachusetts, United States of America; Massachusetts General Hospital and Harvard Medical School, United States of America

## Abstract

**Background:**

Dementia takes decades to develop, and effective prevention will likely require early intervention. Thus, it is critical to identify biomarkers of preclinical disease, allowing targeting of high-risk subjects for preventive efforts. Since telomeres shorten with age and oxidative stress, both of which are important contributors to the onset of dementia, telomere length might be a valuable biomarker.

**Methodology/Principal Findings:**

Among 62 participants of the Nurses' Health Study, we conducted neurologic evaluations, including patient and caregiver interviews, physical exam, neurologic exam, and neuropsychologic testing. We also conducted magnetic resonance imaging (MRI) in a sample of 29 of these women. In these preliminary data, after adjustment for numerous health and lifestyle factors, we found that truncated telomeres in peripheral blood leukocytes segregate with preclinical dementia states, including mild cognitive impairment (MCI); the odds of MCI were 12-fold higher (odds ratio = 12.00, 95% confidence interval 1.24–116.5) for those with shorter telomere length compared to longer telomere length. In addition, decreasing telomere length was strongly related to decreasing hippocampal volume (p = 0.038).

**Conclusions:**

These preliminary data suggest that telomere length may be a possible early marker of dementia risk, and merits further study in large, prospective investigations.

## Introduction

Dementia takes years, if not decades, to develop. It is becoming increasingly clear that effective prevention will require early intervention – that is, prior to the onset of significant neurodegeneration or detectable clinical symptoms. Thus, discovering biomarkers that predict preclinical disease is critical to successful research on lifestyle factors or pharmaceutical agents that can decrease dementia risk [1]. In recent years, telomere length has emerged as a potential marker for biological aging and age-related diseases. Telomeres, repetitive DNA structures that protect the ends of eukaryotic chromosomes, shorten with age. Although neurons are post-mitotic, telomere shortening has been observed in microglia, and regulation of microglial activation may play a important role in the pathogenesis of Alzheimer disease (AD)[2]. Moreover, as a site of high metabolic activity, brain tissue is particularly vulnerable to oxidative damage, and long-term oxidative stress with aging is believed to be an initiating factor in dementia development [3]; since evidence indicates that both age and oxidative damage contribute to telomere shortening [4], telomere length could be a risk marker for dementia in its capacity as a powerful measure of systemic imbalances in oxidative stress and antioxidant defenses. Thus, several lines of evidence indicate that telomere length might be a promising means of evaluating early risk of AD development.

We used the Nurses' Health Study to conduct a pilot study to begin to explore whether telomere length in peripheral blood leukocytes (PBL) might be associated with dementia, especially the early phases marked by mild cognitive impairment (MCI) and hippocampal atrophy.

## Methods

### Nurses' Health Study

The Nurses' Health Study began in 1976, when 121,700 registered female nurses, aged 30 to 55 years, from eleven US states, responded to a mailed questionnaire. To date, 90% follow-up of the cohort has been maintained. For this pilot study, we conducted detailed neurologic evaluations among a sample of participants living in the Boston area. This sample was selected from the 525 Nurses' Health Study subjects in the Boston area who were aged 70 years or older, and who participated in telephone dementia screening [5] from 2005–2006. Briefly, eligible subjects for the neurologic evaluation were all women whose screening indicated possible cognitive impairments, and a random selection of those with no apparent impairments. Of the eligible women, 75% participated in the neurologic exam, and participation rates were similar in those whose screening did and did not indicate impairments. Neurologic evaluation included patient and caregiver interviews, physical exam, neurologic exam, and administration of the Weintraub Activities of Daily Living Scale, Blessed Dementia Scale, and Clinical Dementia Rating Scale. All subjects provided written, informed consent.

Based on the neurologic evaluations, of the 62 women in the study sample, we diagnosed 5 with Alzheimer's disease (by NINCDS/ADRDA and DSM-IV criteria) and 8 with MCI (by Peterson criteria), and 49 who were cognitively intact [6], [7]. We also conducted magnetic resonance imaging (MRI) in a sample of 29 of these women (3 with dementia, 7 with MCI, 19 cognitively intact). We used a 1.5 T Siemens Avanto MRI scanner (Siemens Medical Systems, Iselin, NJ) with high-resolution T_1_-weighted scans. MPRAGE images were used to estimate regional atrophy in the hippocampus (TR = 7.25 msec, echo time TE = 3.0 msec, flip angle = 7°; FOV = 256 mm, matrix = 256×192, 1.33 mm sagitally acquired slices, NEX = 1). Total hippocampal volume was calculated by summing the volumes of the right and left hippocampus, dividing by the intracranial volume, and multiplying by 1000.

As the time of the neurologic exam, we collected blood samples from all 62 participants. To measure PBL telomere length, we extracted genomic DNA from buffy coats using the QIAmp (Qiagen, Chatsworth, CA) 96-spin blood protocol. Relative average telomere length, expressed as the ratio of telomeres to single genes (T/S ratio), was assessed by a modified version of the real-time PCR-based telomere assay [8].

### Statistical Analysis

To quantify the relation of PBL telomere length to odds of dementia and MCI, we used logistic regression models to compute the odds ratios (OR) and 95% confidence intervals (CI), comparing women with shorter telomere length (defined as relative T/S ratio below the median) versus longer telomere length (relative T/S ratio above the median). We also used linear regression models to estimate adjusted mean differences in hippocampal volume associated with each unit increase in relative T/S ratio. In the regression models, we considered the following potential confounding factors: age (continuous years), educational attainment (college degree, master/doctoral degree), cigarette smoking (never or past, current), history of cardiovascular disease (yes, no), high blood pressure (yes, no), high cholesterol (yes, no), and type 2 diabetes (yes, no).

## Results

As expected, women with MCI or dementia were slightly older than controls, although the age distribution in this cohort was relatively narrow, and had less education than controls (Table 1). Blood pressure was somewhat lower in the cases than controls. On average, mean telomere length was progressively shorter in those with MCI or dementia than controls, as was mean hippocampal volume.

**Table 1:** Characteristics of Controls, Cases of Mild Cognitive Impairment (MCI), and Cases of Dementia

	Controls (n = 49)	MCI cases (n = 8)	Dementia cases (n = 5)
Mean age (SD), years	79.2 (2.2)	79.9 (1.3)	80.0 (1.4)
Master's or doctorate degree (%)	8.5	0.0	0.0
Mean systolic blood pressure (SD), mmHg	136.6 (17.3)	136.3 (13.0)	132.0 (17.9)
Mean diastolic blood pressure (SD), mmHg	75.6 (9.4)	73.4 (9.5)	71.8 (12.1)
Mean telomere length (SD), measured as relative telomere/single gene ratio	0.61 (0.14)	0.51 (0.08)	0.49 (0.09)
Mean right hippocampal volume (SD)	2.13 (0.40)	1.86 (0.35)	1.37 (0.68)
Mean left hippocampal volume (SD)	1.93 (0.36)	1.74 (0.24)	1.40 (0.42)

After adjusting for age and educational attainment (Table 2), we found that women with PBL telomere length below the median had a statistically significant, higher odds of dementia or MCI (OR = 9.63, 95% CI 1.73–53.65). Second, we examined telomere length in relation to pre-clinical disease only (Table 1); the odds of MCI were 12-fold higher (OR = 12.00, 95% CI 1.24–116.5) for those with shorter telomere length compared to longer telomere length. Further adjustment for a variety of health and lifestyle factors did not appreciably change any of these results.

**Table 2:** Odds of Dementia or Mild Cognitive Impairment (MCI), According to Telomere Length

	Odds Ratio* (95% confidence interval) for Dementia/MCI	Odds Ratio* (95% confidence interval) for MCI only
Shorter telomere length vs. longer telomere length†	9.63 (1.73–53.65)	12.00 (1.24–116.46)

* Odds ratios are adjusted for age and educational attainment.

† Telomere length measured as relative telomere to single gene ratio in peripheral blood leukocytes. Shorter telomere length was defined as below the median in the population of those without any dementia or mild cognitive impairment.

We also examined the relation of PBL telomere length to hippocampal volume (data not shown in table). We excluded women with dementia from these analyses, so that we could assess telomere length as a possible marker for pre-clinical changes in hippocampal volume. We found that decreasing telomere length was strongly related to decreasing hippocampal volume; after adjusting for age and educational attainment, each unit decrease in relative T/S ratio resulted in a 0.25 mL decrease in hippocampal volume (p = 0.038). Adjustment for a variety of health and lifestyle factors did not alter these findings.

## Discussion

These preliminary data are the first to suggest that shorter PBL telomere length is related to combined dementia/MCI diagnosis, as well as to pre-clinical dementia risk, including both MCI and decreased hippocampal volume. Observed relations were independent of age, educational attainment, cigarette smoking, and various vascular factors, suggesting that PBL telomere length may be a specific marker of dementia.

In particular, our findings for a relation between telomere length and hippocampal volume in those without dementia indicate the possibility that telomere shortening may be a very early indicator of dementia risk. In understanding the magnitude of association we observed between telomere length and hippocampal volume, we compared our results to other studies of hippocampal volume in older subjects. For example, the Rotterdam study reported that the apolipoproteinE e4 allele was related to a 0.21 mL decrease in hippocampal volume [9], while we found that each single 0.1 unit change in the telomere to single gene ratio was related to a 0.25 mL decrease in hippocampal volume (ie, a 0.2 unit change is related to a 0.50 mL decrease; a 0.3 unit change is related to a 0.75 mL decrease, etc).

Limitations of these results should be considered. First, this was a cross-sectional study, thus it cannot be determined whether telomere length predicts dementia or whether dementia leads to telomere shortening. However, in the context of a disease marker, establishing such temporal associations may not be critical. Moreover, our findings were consistent across a variety of disease states, from dementia to MCI to reduced hippocampal volume, suggesting that telomere shortening is an early sign of disease, rather than a later effect of disease onset. Second, the study sample was very small, and, although findings were statistically significant, the confidence intervals were large, indicating that the data were compatible with a wide range of associations. Thus, these should only be considered as preliminary findings, and interpreted with caution. Nonetheless, it is reassuring that we observed robust associations between telomere length and multiple cognitive outcomes. In addition, the limited literature generally supports our findings. In a small, prospective study of 195 stroke survivors, telomere length in peripheral blood mononuclear cells (PBMC) at baseline was significantly related to both risk of developing dementia over two years (OR = 0.1, 95% CI 0.0–0.8) and to decline on the MMSE, with a mean decrease of 0.77 points on the MMSE for each 1000 bp decrease in telomere length (p = 0.04) [10]. Among 257 older Hispanic, Caucasian and African-American individuals, in a nested case-control study, PBL telomere length was shorter in those with AD than controls (mean = 0.46 vs 0.52, p<0.03, respectively) [11]. Finally, in a cross-sectional study of PBL telomere length in 559 older subjects, there was a significant relation between PBL telomere length and verbal fluency (p = 0.02), although not other cognitive systems [12].

Overall, these preliminary data and limited existing studies indicate a possible role for PBL telomere length in identifying older persons with high risk of dementia. As an easily-measured and non-invasive biomarker, further research regarding these relations in large, prospective studies is needed.
